# A Network of Circular RNA and MicroRNA Sequencing Provides Insights into Pigment Deposition of Changshun Blue Eggshell Chickens

**DOI:** 10.3390/genes15060812

**Published:** 2024-06-19

**Authors:** Siyu Chen, Mengqiao Zhao, Kecheng Chen, Jiaming Xu, Hua Li

**Affiliations:** Guangdong Provincial Key Laboratory of Animal Molecular Design and Precise Breeding, Key Laboratory of Animal Molecular Design and Precise Breeding of Guangdong Higher Education Institutes, School of Life Science and Engineering, Foshan University, Foshan 528231, China

**Keywords:** chickens, circRNAs, color deposition, miRNA, uterus

## Abstract

Eggshell color plays important biological roles and attracts the attention of both egg retailers and researchers. However, whether non-coding RNAs are involved in pigment deposition among different eggshell colors remains unknown. In this study, RNA sequencing was used to analyse the uterine gland transcriptome (CircRNA and miRNA) of Changshun chicken blue-shell hens producing four different eggshell color eggs including dark blue PK(DB) and light blue (LB), dark brown and greenish (between blue and pink, DP) and pink (p). We found that *miR-192-x*, targeting *SLC16a7*, was expressed in DB, DP, and LB groups compared with the PK group, which indicates that *miR-192-x* may play a role in the blue eggshell color. KEGG and GO analyses showed that the “metabolic pathways” with targeted genes such *BLVRA* and *HMOX1* were detected in dark and light blue color eggshell chickens, which confirms the different ratios of biliverdin and *HO-1* involved in the deposition of blue color. As annotated by connectivity analysis, *RASGRF1* and *RASGRF2*, belonging to the *RASGRF* family, are involved in the *Ras* signaling pathway, which plays an important role in cell growth, differentiation, metastasis and apoptosis. Our findings enrich the database of circRNA, miRNAs and genes for chicken uterine tissue, which will be useful in accelerating molecular selection for blue eggshell color layers.

## 1. Introduction

Internationally, consumer preferences for eggshell color, including unique options such as green eggs, exhibit variance based on regional and cultural factors. For instance, in certain regions of Asia and Europe, eggs with colored shells are seen as distinct and potentially more attractive due to their perceived health advantages and natural appeal [1,2]. In China, preferences for eggshell color also differ, with white and brown eggs being more prevalent. Green eggs, typically laid by specific breeds such as Dongxiang, Lushi chickens and Changshun blue eggshell chickens, are less widely available on the market [2]. Nevertheless, some consumers favor these eggs for their distinctive color and perceived health benefits, while the actual nutritional differences are minimal [3]. Although still a niche market compared to the predominant white and brown eggshell sectors, the demand for green eggs is on the rise [4].

The eggshell pigment is generally formed from the protoporphyrin, biliverdin and zinc chelate of biliverdin, which results in various eggshell colors. The degree of color in an eggshell is mainly determined by the total amount and proportion of pigment [5]. For birds, brown, white, pink and blue are the most common eggshell colors. Protoporphyrin is an antioxidant [6] and a direct precursor of heme, which is involved in the color deposition in pink, brown or yellow eggshells [7]. Biliverdin has antioxidant activity and is a by-product of hemoglobin decomposition, while biliverdin IX, and biliverdin IX zinc chelate are associated with blue and green-blue eggshell colors [8]. As long as these pigments are not found in the uterine gland, the eggshell color turns out to be white [9,10].

Recently, a few studies regarding protein-coding genes have been carried out to illustrate the genetic mechanisms of pigment deposition in poultry. It is generally accepted that the solute carrier organic anion transporter family member 1B3 (*SLCO1B3*) gene displays functions relating to the regulation of pigment deposition for the blue eggshell phenotype in both domestic and foreign chickens [11]. So far, with the development of omics technology, long non-coding RNA (lncRNA) is deemed to be critical in numerous biological processes including transcriptional regulation [12,13], the progression of cancer [14], and human genetic disorders [15,16]. A previous study has shown that *ALAS1*, *TAL* and *SLC13A5* may be key genes regulating biliverdin synthesis by integrating microRNA (miRNA) and messenger RNA (mRNA) sequencing in duck oviduct shell glands [17]. In our previous study, we also reported that lncRNAs exert similar functions with mRNA in color formation by modulating pigment disposition in Changshun blue eggshell chickens [18]. In addition, circular RNAs (circRNAs), as a novel category of endogenous non-coding RNAs (ncRNAs), were identified during intron self-splicing from mitochondrial RNAs, ribosomal RNAs, and transfer RNAs. Increasing evidence indicates that circRNAs play an essential role in controlling transcriptional regulation by maintaining homeostasis [19,20,21]. CircRNAs are known to bind with miRNA to prevent mRNA translation, and interact with RNA-associated proteins or influence gene expression by regulating gene splicing or mRNA levels [22]. Nevertheless, little is known as to whether circRNAs or miRNAs contribute to pigment deposition, and the underlying mechanism needs to be further investigated.

In this study, circRNA and miRNA sequencing was used to determine the uterine transcriptome, and a network of these two ncRNAs was developed to enable better understanding of color formation in Changshun chicken eggs. Our objective was to identify and profile ncRNAs in chicken shell glands and obtain candidate circRNAs, miRNAs, and pathways related to blue eggshell deposition in poultry.

## 2. Materials and Methods

### 2.1. Animals and Sample Collection

Changshun blue eggshell layers were obtained from a breeding farm, owned by Tiannong Corporation (Guizhou, China). Thirty days were spent observing 331 layers from the 12th generation preserved population that were housed at 210 days old. The approximate laying time of each group of hens was observed and recorded hourly from 8:00 am to 2:00 pm daily for three consecutive days. Three layers consistently producing dark blue (DB) and light blue (LB) eggs, and four layers producing dark brown and greenish (between blue and pink, DP) eggs were chosen during the peak laying period (about 210 days of age). In addition, four layers producing pink (PK) shell eggs were used as the study control group. The uterine shell gland tissues were taken from birds slaughtered at 7 h after egg laying, which was monitored by cameras hanging on opposite cages. The shell gland from each bird was collected and immediately stored in dry ice and then at −80 °C until being further processed. All experiments were performed using protocols approved by the Animal Care Committee of Foshan University (approval ID: FOSU#080).

### 2.2. RNA Extraction, Strand-Specific Library Construction and Sequencing

Following the extraction of total RNA using the Trizol reagent kit (Invitrogen, Carlsbad, CA, USA), a nucleic acid protein analyzer was used to analyze the RNA total concentration and optical density (OD) of the samples at wavelengths of 260 nm and 280 nm. The OD260/280 ratio needed to be within the range of 1.8–2.0 for the samples to meet the required specifications. The data of quality analysis of total RNA can be found in Supplementary Table S1. Next, polyacrylamide gel electrophoresis (PAGE) was used to separate the RNA molecules within a size range of 18–30 nt. The 36–48 nt RNAs were then enriched after the addition of 3′ adapters. The 5′ adapters were then ligated to the RNAs. The ligation products were reverse transcribed by Polymerase Chain Reaction (PCR) amplification and the 140–160 bp size PCR products were used to generate DNA (cDNA) libraries, which were sequenced using an Illumina HiSeq X10 platform at Gene Denovo Biotechnology Co., Ltd. (Guangzhou, China). Then, each sample was diluted to a concentration of 300 ng/µL using DEPC. The Vazyme III RT SuperMix for qPCR (+gDNA wiper) kit was utilized for the reverse transcription synthesis of cDNA, with careful pipetting and mixing involved. The reaction was carried out at 42 °C for 2 min, followed by the addition of 4 µL of 5× HiScript III qRT SuperMix at 37 °C for 15 min and a final step at 85 °C for 5 s.

### 2.3. Differential Expression Analysis of mRNA, miRNA and circRNA

After the total RNA was extracted, rRNAs were removed to retain mRNAs and ncRNAs. The enriched mRNAs and ncRNAs were fragmented into short fragments by using a fragmentation buffer and reverse transcripted into cDNA with random primers. Second-strand cDNA was synthesized by DNA polymerase l, RNase H, dNTP (dUTP instead of dTTP) and a buffer. Next, the cDNA fragments were purified with the QiaQuick PCR extraction kit, end repaired, mixed with poly(A), and ligated to Illumina sequencing adapters. Then, UNG (Uracil-N-Glycosylase) was used to digest the second-strand cDNA. The digested products were size selected by agarosegel electrophoresis, PCR amplified, and sequenced using illumina HiSeq^TM^ 4000 by Gene Denovo Biotechnology Co., Ltd. (Guangzhou, China). The expression levels of mRNAs were quantified using FPKM (fragments per kilobase of transcript per million mapped reads). Using the DESeq2 [23] (https://bioconductor.org/, accessed on 24 July 2020) software package, differentially expressed circRNAs were first screened using the FDR-adjusted *p*-value. However, there were few differentially expressed circRNAs. Thus, the genes that exhibited an FDR < 0.05 and log2(fc) > 1 were identified as significantly differentially expressed [24,25,26]. TPM (transcripts per million reads) was used to calculate the expression of miRNAs, and edgeR (http://www.rproject.org/, accessed on 24 July 2020) software was used. RPM (back-spliced Reads Per Million mapped reads) was used to calculate the expression of circRNA and edgeR [27] (https://bioconductor.org/, accessed on 24 July 2020) software was also used for analysis. A threshold of *p* ≤ 0.05 and log2(fc) > 1 determined significant differential expression. In order to identify distinct anchor positions within a splice site, 20mers from both ends of the unmapped reads were extracted and aligned to the reference genome (Ensembl_release92, Galllus_gallus-5.0). Anchor reads that aligned in the reversed orientation (head-to-tail) indicated circRNA splicing and then were subjected to find_circ [28] to identify circRNAs. The anchor alignments were then extended to ensure that the complete reads aligned and the breakpoints were flanked by GU/AG splice sites. A candidate circRNA was used if it was supported by at least two unique back-spliced reads in at least one sample. Clean tags were then searched against the miRBase database (Release 22) to find known miRNAs (existing miRNAs). Not all chicken miRNA sequences are represented in the miRBase database, so alignment with miRNAs from other species was also a dependable way to identify known miRNAs. All of the unannotated tags were aligned with the reference genome (Ensembl_release92, Galllus_gallus-5.0). According to their genome positions and hairpin structures predicted by software miRDeep2 [29] (https://github.com/rajewsky-lab/mirdeep2, accessed on 24 July 2020), novel miRNA candidates were identified.

### 2.4. Construction of DEcircRNA-DEmiRNA-DEmRNA Regulatory Network

Target gene prediction was performed using miRanda (http://www.microrna.org/, accessed on 26 July 2020) and TargetScan (https://www.targetscan.org/, accessed on 26 July 2020) software with DEmiRNAs. Spearman’s rank correlation coefficients (SCCs) of miRNA and candidate competing endogenouse RNAs were calculated for the above target gene pairs, and target gene pairs with correlation coefficients ≤ −0.5 were screened. Following the calculation of the SCC for the expression between the competing endogenous RNA (ceRNA) pairs acquired in the preceding step, the ceRNA pairs with a correlation coefficient of more than 0.5 were selected as potential ceRNA pairs. ceRNAs with a P value less than 0.05 were screened using the hypergeometric distribution test. The Cytoscape v3.6.0 (http://www.cytoscape.org/, accessed on 27 July 2020) software was used to build a network map for the interaction between circRNA-miRNA-mRNA. The ceRNA connectivity analysis was performed, and in the ceRNA regulatory network, the higher the number of miRNA molecules with miRNA- or cicrRNA-targeting regulatory relationships, the higher the connectivity. Nodes with high connectivity often have important biological significance.

### 2.5. Quantitative Real-Time Polymerase Chain Reaction (RT-qPCR)

In light of potential functional importance, RT-qPCR was performed on DEmiRNAs selected from each comparison. Based on the DEmiRNA sequences obtained by sequencing, primers were designed at Sangon Biotech (www.sangon.com/primerDesign, accessed on 29 July 2020). The primer sequence was designed using Primer sequence 5.0 and synthesized by the Shanghai Bioengineering company (Supplementary Table S2). Glyceraldehyde-3-phosphate dehydrogenase (*GAPDH*) was selected as a reference gene. The OD 260/OD 280 ratio was measured for each RNA sample and RNA integrity was analyzed by agarose gel electrophoresis. RNA was confirmed to be of high quality and was then reverse transcribed to cDNA using TRUEscript RT MasterMIX in a 20 μL volume containing l000 ng RNA and RNase free to 16 μL, 4 μL 5 × TRUE RT MasterMix under the following conditions: 42 °C for 10 min (Aidlab, PC5801, Hangzhou, China) for RT-qPCR analyses. RT-qPCR was conducted on qTOWER 2.2 touch (Analytik, Jena, Germany) in a 20 μL volume containing 10 μL SYBR × Premix Ex Taq (Aidlab, PC3302, China), 0.5 μL of each forward and reverse primer (10 μM), 1 μL of cDNA, and 8 μL ddH_2_O under the following conditions: 95 °C for 15 min; 95 °C for 10 s, annealing for 20 s and 72 °C for 20 s for 40 cycles. Each amplification was performed for three control replicates and three case replicates. The amplification efficiencies were close to 100%, using the 2^−ΔΔCt^ method for calculating the relative gene expression levels of a sample. ΔΔCt = (target gene Ct in the experimental group—reference gene Ct in the experimental group)—(target gene in the control group—reference gene in the control group). U6 can be used for miRNA or cirRNA normalization as shown in previous research [30], and was used as an internal reference gene. MiRNA First Strand cDNA Synthesis (Stem-loop Method) (Sangon Biotech, Shanghai, China) was used for cDNA synthesis. The qPCR machine was programmed to be 16 °C for 30 min, then 37 °C for 30 min, and finally 85 °C for 5 min. RT-qPCR was performed on each sample in triplicate using the ChamQ Universal SYBR qPCR Master Mix (Sangon Biotech, China) on QuantStudio5 (Applied Biosystems, Waltham, MA, USA) in a 20 μL reaction volume. The program settings were as follows: pre-denaturation at 95 °C for 30 s, 40 cycles of reaction at 95 °C for 10 s, melting curve at 95 °C for 15 s, 60 °C for 60 s, and 95 °C for 15 s. Melting curve analysis was used to check the amplification specificity. The relative expression of target gene transcripts was calculated using the comparative Ct method (2^−ΔΔCt^) and subjected to statistical analysis with SPSS software (version 19) (https://www.ibm.com/, accessed on 20 May 2020).

## 3. Results

### 3.1. Overview of Sequencing and Identification of miRNA in Chicken Uterus

Following quality control, an average of 90.4% of the total high-quality clean reads were mapped to Galgal 5.0, GenBank and Rfam, Exon and Repeat (Supplementary Table S3). Accordingly, 524 miRNAs, known by blasting against the known chicken miRNAs in the ALDB database, were captured, with 276 of these miRNAs expressed in all four groups. In addition, 16, 12, 26 and 18 miRNAs were specifically expressed in DB, DP, LB, and PK groups, respectively (Table 1). In addition, 53 of 142 new miRNAs were seen to be expressed in all four groups (Table 1).

Between the DB vs. PK group, there were 31 significantly differentially expressed miRNAs (DEmiRNAs) identified, among which 10 were upregulated, and 21 were downregulated in the DB group compared to the PK group. Between the LB vs. PK group, 53 DEmiRNAs were identified with 36 upregulated and 17 downregulated in the LB group as compared to the PK group. Between the DP vs. PK group, 18 DEmiRNAs were identified, with 10 upregulated and 8 downregulated DEmiRNAs in the former group and latter group, respectively. Between the DP vs. DB group, 41 DEmiRNAs were obtained, of which 23 were upregulated and 18 were downregulated in the DB group compared to the DP group. Additionally, only *miR-2995-x* and *novel-m0026-5p* were significantly expressed in the four comparison groups, of which the highest expression was the DB vs. PK group. *miR-192-x* was seen to be expressed in the DB vs. PK, LB vs. PK, and the DP vs. PK group. The top three up and downregulated miRNAs of significantly differentially expressed miRNA in all the four-comparison groups are shown in Table 2.

### 3.2. Functional Enrichment Analysis of Differently Expressed miRNA and Targeted Genes

Furthermore, based on these miRNAs, Miranda and TargetScan were used to predict target genes (Table 2). Between the DB vs. PK group, there were 2995 target mRNAs identified, of which 723, 1473 and 297 target mRNAs were found by the upregulated *miR-2995-x*, *novel-m0026-5p* and gga-miR-3528, while 162, 220, and 220 target mRNAs were obtained by the downregulated miR-423-y, novel-m0066-5p and novel-m0067-5p. Between the LB vs. PK groups, 4555 genes were identified, of which 629, 723 and 1493 target genes were identified by the upregulated miR-224-x, *miR-2995-x* and *novel-m0026-5p*, while 556, 556 and 618 target genes were identified by the downregulated gga-miR-2984-3p, gga-miR-6552-3p and gga-miR-6552-5p. Between DP vs. PK, 4245 target genes were found, of which 549, 594 and 723 genes were predicted by the upregulated novel-m0093-3p, novel-m0142-3p and *miR-2995-x*, while 471, 1420 and 488 target genes were predicted by the downregulated *miR-484-x*, gga-miR-6544-5p and novel-m0096-3p. Between the comparison of the DB vs. DP group, 3614 genes were identified, with 220, 220 and 300 target genes predicted by the upregulated novel-m0066-5p, novel-m0067-5p and gga-miR-6516-3p and 875, 579 and 1420 genes predicted by gga-miR-217-5p, novel-m0136-5p and gga-miR-6544-5p.

**Figure 1:**
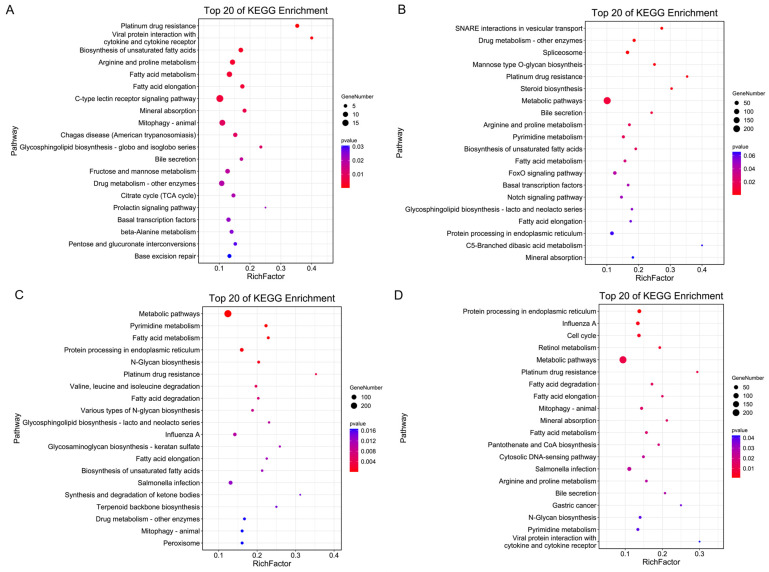
KEGG pathway enrichment analysis of highly expressed differential miRNA target genes with the top twenty enrichment scores. (**A**) DB vs. PK. (**B**) DP vs. PK. (**C**) LB vs. PK. (**D**) DB vs. LB. The *Y*-axis label represents the pathway and the *X*-axis label represents the rich factor. The colour and size of the bubble represent enrichment significance and the amount of differentially expressed genes enriched in the pathway, respectively. The analysis includes DB, DP, LB, and PK mean chickens producing dark blue, dark brown and greenish, light blue, and pink eggshell eggs, respectively.

We then performed gene ontology (GO) enrichment and Kyoto Encyclopedia of Genes and Genomes (KEGG) enrichment analysis based on these target genes. In each comparison group, GO enrichment analysis revealed that these target genes were significantly enriched in the cellular process, metabolic process, cell, cell part, binding and catalytic activity (Supplementary Figure S1A–D). Bubble charts were used to represent the top 20 pathways in the DB vs. PK group (Figure 1A), LB vs. PK group (Figure 1B), DP vs. PK group (Figure 1C), and DB vs. DP group (Figure 1D), respectively. In the DB vs. PK group, KEGG pathways were mainly involved in platinum drug resistance, viral protein interaction with cytokine and cytokine receptors, biosynthesis of unsaturated fatty acids, mineral absorption, fatty acid metabolism; arginine and proline metabolism (Figure 1A). In the LB vs. PK group, KEGG pathways were mainly enriched in SNARE interactions in vesicular transport, drug metabolism-other enzymes, mannose type O-glycan biosyntheis, platinum drug resistance, steroid biosynthesis. (Figure 1B). In the LB vs. PK group, KEGG pathways were involved in metabolic pathways, pyrimidine metabolism, fatty acid metabolism, protein processing in the endoplasmic reticulum; platinum drug resistance (Figure 1C). In the DB vs. DP group, KEGG pathways were involved in protein processing in the endoplasmic reticulum, cell cycle, retinol metabolism and metabolic pathways (Figure 1D).

### 3.3. Overview of Sequencing and Identification of circRNA in Chicken Uterus

In total, 861,368,284 reads were obtained. After quality control, the averages of effective reads of Q20 and Q30 were 98.23% and 94.56%, respectively (Supplementary Table S4). More than 0.71% of rRNA was removed from these clean reads of each sample. Afterwards, these reads were mapped to the chicken genome, and an 83.89% mapping rate was obtained (Supplementary Table S5).

In total, 13235 circRNAs were identified, of which there were 7931, 7816, 8791 and 8674 circRNAs in the DB, DP, LB and PK groups, respectively, and 6659 circRNAs were found in all four groups. The length of these circRNAs averaged around 300–400 bp, 400–500 bp and 3000 bp, and the circRNAs were located on chromosomes 1, 2, 3, 4 and Z. Accordingly, these circRNAs were annotated to 5940 annot_exons, 1914 antisense, 1870 exon_intron, 1723 intergenic, 1138 intronic and 650 one_exon (Supplementary Figure S2).

Between the DB vs. PK group, there were 129 differentially expressed circRNAs identified, of which 70 were upregulated and 59 were downregulated. Between the LB vs. PK group, there were 122 differentially expressed circRNAs with 50 upregulated and 70 downregulated circRNAs obtained. Between DP vs. PK, 121 differentially expressed circRNAs were identified, of which 50 were upregulated and 71 downregulated. Novel_circ_001519, novel_circ_012603, novel_circ_001808, novel_circ_001461, novel_circ_003137, novel_circ_011869, novel_circ_006235, novel_circ_006931, and novel_circ_005940 were found to be expressed in all three comparisons.

### 3.4. Functional Enrichment Analysis of Differentially Expressed circRNA and Target Genes

**Figure 2:**
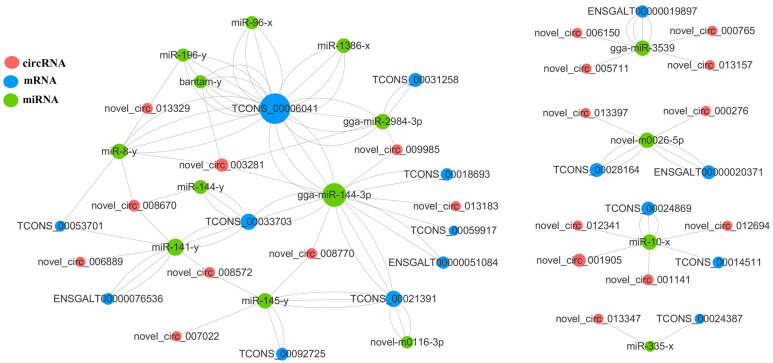
CeRNA networks. CeRNA networks were constructed based on identified circRNA–miRNA and miRNA–mRNA interactions.

The target mRNAs sourced from circRNA were further used for the GO and KEGG analyses (Figure 2). Between the DB vs. PK group, 129 target DEGs were assigned to 201 GO terms (*p* < 0.05), 122 target DEGs were assigned to 261 GO terms in the DB vs. PK group, 121 target DEGs were assigned to 262 GO terms in the DB vs. PK group, and 122 target DEGs were assigned to 249 GO terms in the DB vs. PK group (Supplementary Figure S2).

### 3.5. Construction of the ceRNA Network of Blue-Shell Egg

**Figure 3:**
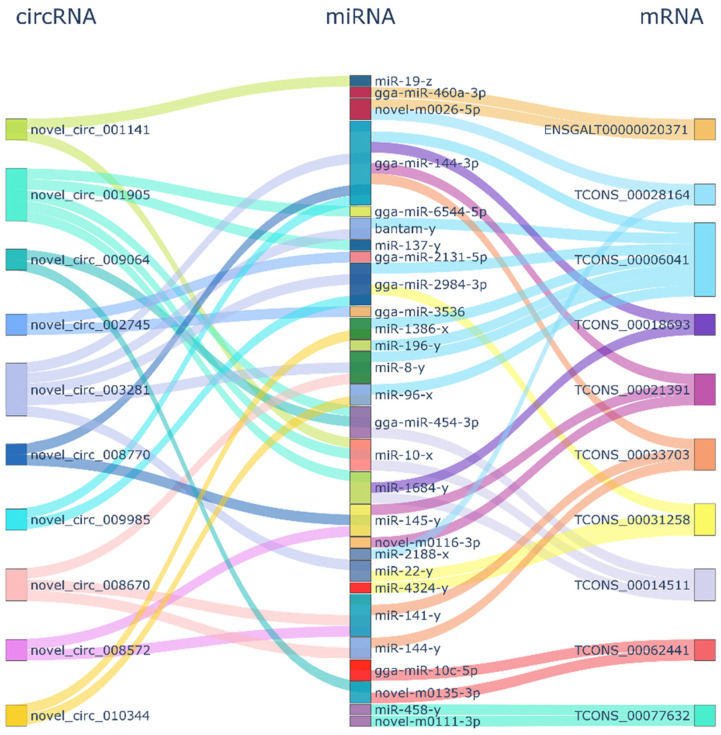
CircRNA-miRNA-mRNA connectivity.

The ceRNA regulatory network contained 63 circRNA-miRNA-mRNA pairs and included 45 circRNAs, 38 miRNAs, and 37 mRNAs (Figure 3). For the top 10 mRNAs and circRNAs in connectivity, a Sankey diagram of their relationship with miRNA-targeting regulations was drawn (Figure 3). The mRNA in the figure corresponds to the symbol number to find six genes, *TCONS_00006041(BBX)*, *TCONS_00021391-GPC6*, *TCONS_00077632-ZBTB44*, *TCONS_00062441-ICA1*, *TCONS_00018693-GRIK1* and *TCONS_00028164-RASGRF1*.

### 3.6. Validation of DEmiRNAs by RT-qPCR

**Figure 4:**
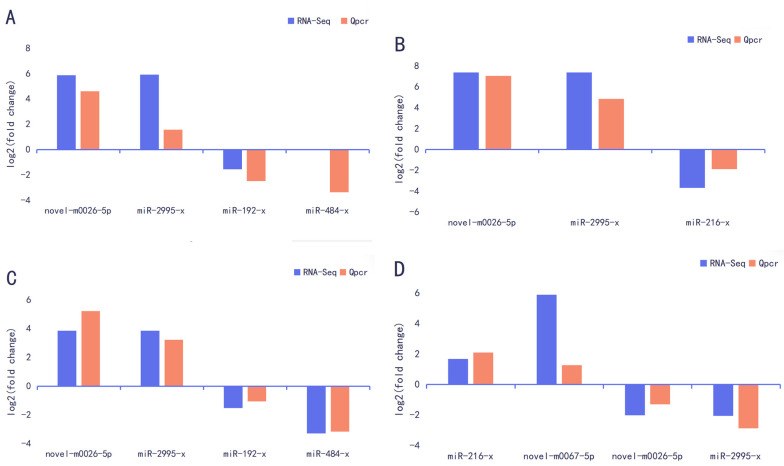
RT-qPCR validation of differentially expressed miRNAs expression. (**A**) DB vs. PK. (**B**) DP vs. PK. (**C**) LB vs. PK. (**D**) DB vs. LB.

The DB vs. PK group, LB vs. PK group, DP vs. PK group, and DB vs. PK group were used to measure the relative expression of DEmiRNAs using RT-qPCR. The relative expression (log2FC) of these DEmiRNAs was similar between the two approaches, although some quantitative differences were found between the RT-qPCR and RNA-seq analytical platforms (Figure 4). Therefore, the RNA-seq results were reliable and could be used for bioinformatics analysis.

## 4. Discussion

There is a growing trend in the market towards the consumption of organic and free-range eggs, particularly those with brown or green shells. These eggs are often perceived by consumers as being associated with higher animal welfare and fewer chemicals, reflecting a broader shift towards sustainable and ethically produced food. In China, there is a vast array of native poultry, including 108 indigenous chicken breeds. Some of these local hens lay eggs with blue shells, which are highly favored by the majority of consumers. These blue eggs are considered precious due to their rarity, and are believed to be more flavorful than white or brown eggs as they come from native birds. Blue eggs have a higher egg white height, Hastelloy units, and calcium and phosphorus content compared to white eggs [31]. The higher Hastelloy unit indicates better protein quality in blue eggs, leading to the conclusion that they are of superior quality compared to white eggs. Additionally, green eggs have been found to have significantly lower cholesterol content than brown eggs [32]. As a result of consumer demand, there has been a deliberate selection process in place for breeders to produce layers that lay blue eggs over the past few decades.

In this study, an miRNA and circRNA network was constructed to illustrate the role of non-coding RNA in the color formation of blue eggshell chickens. To date, miRNAs have been demonstrated to exert essential roles in pigment deposition in several human and animal studies. For example, the significantly upregulated miRNA-21, and downregulated miRNA -320 and miRNA-494 were found to be associated with skin in human melanoma [33]. Furthermore, the hair color of alpacas is determined by the production of melanin in melanocytes, and the target genes are known to be regulated by miRNA [34]. Also, in chickens, it is known that some miRNAs and their target genes play an important role in muscle melanin production [35]. In the current study, small RNA sequencing was performed on the uterine shell gland of four different eggshell color birds. In total, 666 miRNAs were obtained, of which there were 524 known miRNAs and 142 new miRNAs. Subsequently, *miR-192-x* was found to be expressed in DP vs. PK, DB vs. PK and LB vs. PK, which indicates that it may play a role in blue eggshell color deposition. Interestingly, miR-192 has demonstrated to be associated with the mechanism of tilapia response to carbonate alkalinity stress, and *SLC* solute vector family member *SLC16*a7 is the target gene of miR-192 [36]. In addition, miR-192-5p is highly expressed in endometrial epithelial cells in the non-receptive state and is involved in maintaining epithelial cell polarity and anti-adhesion properties on the cell surface [37,38]. Notably, *SLC* genes are well known to be responsible for blue eggshell deposition in chickens [18]. Thus, we speculate that *miR-192-x* is a candidate miRNA regulating blue eggshell color regardless, meaning that the underpinning mechanism should be further investigated.

We came to the following conclusions based on the target genes predicted by three of the most differentially up and downregulated miRNAs. The GO terms were similar between four comparisons, which are generally consistent with enrichments in duck shell glands [1], indicating the reliability of these data. The pathway “platinum drug resistance” and target gene *SLC31A1* were significant across the DP vs. PK, LP vs. PK and BP vs. PK comparisons, but not between the DP vs. LP group. In addition, the “mineral absorption” with the target *SLC* family genes was also found to be enriched in dark blue and pink eggshell chicken groups. These results again provide evidence for the possible role of miRNA in regulating blue eggshell color. Interestingly, the “metabolic pathways” with target genes such *BLVRA*, and *HMOX1* were reported in dark and light blue color eggshell chickens. As is known, protoporphyrin reponds to ferrous ions that are haem via iron chelatase, and then degrades into biliverdin using haem oxygenase 1 (*HO-1*) activity. Further, biliverdin reductase A (*BLVRA*) converts bilirubin into bilirubin [13]. Our results also confirm that different ratios of biliverdin and *HO-1* are involved in the deposition of blue color.

Generally, the vast majorities of circRNAs are often identified in the cytoplasm, and are stable with relatively long half-lives [39]. circRNAs are demonstrated to exert functions in regulating the growth and progression of human cancer, controling the intracellular localization, and tumor autophagy. To date, circRNA studies regarding chicken have been mainly focused on bone growth [40], adipocyte differentiation [41], and follicular development, while the color deposition of chicken eggshell has not yet been reported. In our study, 13235 circRNAs were identified, which is less than the number of circRNAs in a previous study related to follicular development [42]. Accordingly, the ratio of exon, antisense chain, exon–intron, intergenic region, intron and single exon groups is very close to that of a study reporting preadipocyte differentiation in chicken [17].

In terms of biological processes of GO terms, the four comparison groups are mainly focused on metabolic and cellular processes. In terms of cellular components, the four comparator groups were mainly cells, cellular components and organelles. The GO enrichment secondary entries of circRNA-derived genes are similar to those of mRNA and miRNA, indicating the associated genes of mRNA, miRNA and circRNA, which were mainly linked to metabolic and cellular activities by participating and binding activity in cells and cellular components. Furthermore, with regard to the KEGG pathway, “ferroptosis” and the “PPAR signaling pathway” were enriched in both the DB vs. PK and DP vs. PK groups. Heme oxygenase metabolizes heme to produce carbon monoxide, biliverdin and ferric ion. Catalyzing the high expression of unsaturated fatty acids in the cell membrane with the existence of ferrous iron or ester oxygenase may be the possible mechanism of the ferroptosis pathway, which in turn promotes liposome peroxidation and cell apoptosis [43,44]. The concentration of ferric ion affects the efficiency of heme synthesis, which in turn affects the accumulation of protoporphyrin in the epithelial cells of the eggshell gland [17]. The PPAR signaling pathway is associated with lipid metabolism in goldfish [45]. Most of the RNAs in our study were enriched in substance metabolism and transport. Due to their stability, specificity and sensitivity, circRNAs have potential value for eggshell pigment deposition and deserve further investigation.

In the network of circRNA-miRNA-mRNA, various repeated miRNAs were found within comparison groups, i.e., *novel-m0026-5p* repeated four times, and *gga-miR-2984-3p* repeated once. All of these are the key targets in miRNA research. Among them, novel_circ_013347-miR-335-x-TCONS_00024387/DUOXA1 and DUOXA1 belong to the NOX protein family, which are membrane-integrated proteins that share a similar core module responsible for catalyzing redox reactions, consisting of a transmembrane region bound by a double heme molecule and an intracellular dehydrogenase structural domain [46,47]. In contrast, biliverdin is the main final metabolite of heme. This network chain, from its composition to its function, is related to eggshell color and needs further investigation. Six genes, including *BBX*, *GPC6*, *ZBTB44*, *ICA1*, *GRIK1* and *RASGRF1,* were annotated by connectivity analysis. *RASGRF1* and *RASGRF2* belong to the *RASGRF* family and are involved in the *Ras* signaling pathway, which exerts significant effects on development, metabolic and apoptosis [48]. Other genes have scarcely been reported in chickens, but in humans, GPC6 was identified as a biomarker for melanoma metastatic progression [49]. In addition, *ICA-1* decreased migration and melanoma cell invasion [50]. We speculate that although the connectivity in the ceRNA network is high, how the circRNAs exert functions on the greenshell trait still needs further investigation.

## 5. Conclusions

In summary, with respect to transcriptome sequencing of miRNA and circRNA in Changshun chicken shell glands, *miR-192-x*, *miR-2995-x*, and *novel-m0026-5p* are presumed to be related to the regulation of the eggshell’s green color. *Novel-m0026-5p*, *miR-192-x* and *gga-miR-2984-3p* were found as the differential miRNAs in the circRNA-miRNA-mRNA network. Our results provide a catalog of chicken uterine circRNAs and genes worthy of further studies to understand their roles in the selection of blue eggshell color layers.

## Figures and Tables

**Table 1:** Known and new miRNAs.

Samplings	Known miRNAs	New miRNAs
All	524	142
DB1	375	105
DB2	377	110
DB3	373	99
LB1	379	110
LB2	348	94
LB3	363	106
DP1	377	112
DP2	358	113
DP3	364	105
DP4	406	111
PK1	367	105
PK2	384	112
PK3	371	112

DB, DP, LB, and PK mean chickens producing dark blue, dark brown and greenish, light blue, and pink eggshell eggs, respectively.

**Table 2:** Significantly differentially expressed miRNAs.

Comparison Groups	miRNA	log2(fc)	Expression Change	Target Genes
DB vs. PK	miR-2995-x	5.91	up	723
	novel-m0026-5p	5.87	up	1473
	gga-miR-3528	4.21	up	297
	miR-423-y	−6.51	down	162
	novel-m0066-5p	−6.65	down	220
	novel-m0067-5p	−6.65	down	220
LB vs. PK	miR-224-x	8.72	up	629
	miR-2995-x	7.37	up	723
	novel-m0026-5p	7.37	up	1473
	gga-miR-2984-3p	−5.07	down	556
	gga-miR-6552-3p	−7.4	down	556
	gga-miR-6552-5p	−8.49	down	618
DP vs. PK	novel-m0093-3p	5.32	up	549
	novel-m0142-3p	4.43	up	594
	miR-2995-x	3.84	up	723
	miR-484-x	−3.28	down	471
	gga-miR-6544-5p	−4.51	down	1420
	novel-m0096-3p	−5.14	down	488
DB vs. DP	novel-m0066-5p	5.88	up	220
	novel-m0067-5p	5.88	up	220
	gga-miR-6516-3p	5.17	up	300
	gga-miR-217-5p	−3.69	down	875
	novel-m0136-5p	−4.4	down	579
	gga-miR-6544-5p	−5.09	down	1420

DB, DP, LB, and PK mean chickens producing dark blue, dark brown and greenish, light blue, and pink eggshell eggs, respectively.

## Data Availability

The data presented in this study are available at the National Center for Biotechnology Information (NCBI—https://www.ncbi.nlm.nih.gov, accessed on 12 July 2021), under the following accession numbers: BioProject PRJNA1029363; submission SUB13907768.

## Supplementary Materials

The following supporting information can be downloaded at: https://www.mdpi.com/article/10.3390/genes15060812/s1, Figure S1: Differential expression of miRNAs.; Figure S2: GO analysis of highly expressed differential miRNAs target genes.; Table S1: Quality analysis of total RNA.; Table S2: Primers for miRNA RT-qPCR.; Table S3: Small RNA sequencing data quality filtering and annotation.; Table S4: The quality and filtration statistics of sequencing data.; Table S5: Contrast ribosomes and chicken genomes.
