# Supplementary figures and images for: A Network of Circular RNA and MicroRNA Sequencing Provides Insights into Pigment Deposition of Changshun Blue Eggshell Chickens
Source: Genes (Basel). 2024 Jun 19;15(6):812. doi: 10.3390/genes15060812 (PMC11202489; doi:10.3390/genes15060812)

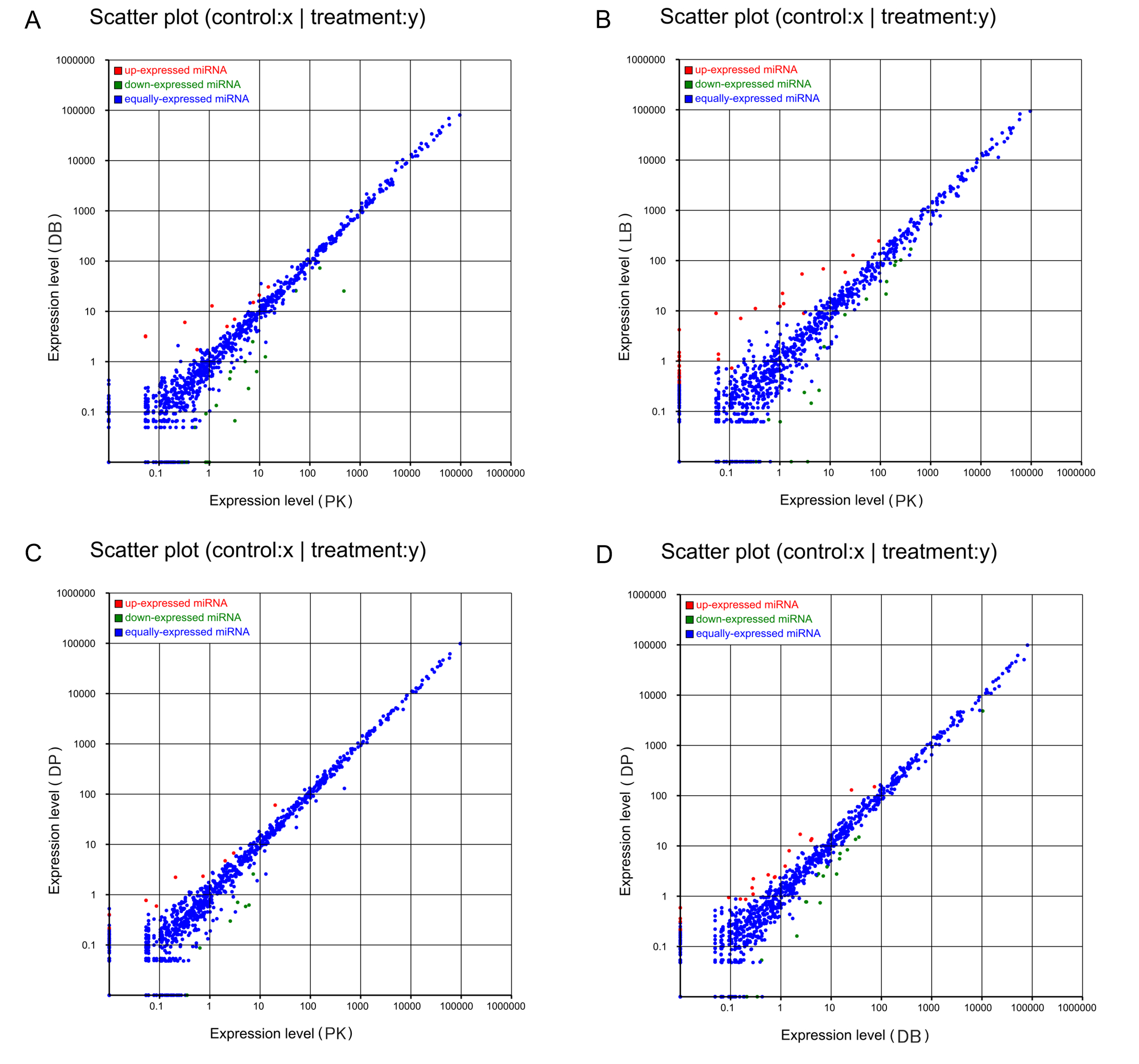

Supplement: Supplementary file 1 [file genes-15-00812-s001.zip › SM Figure/SM Figure S1.Differential expression of miRNAs..jpg]

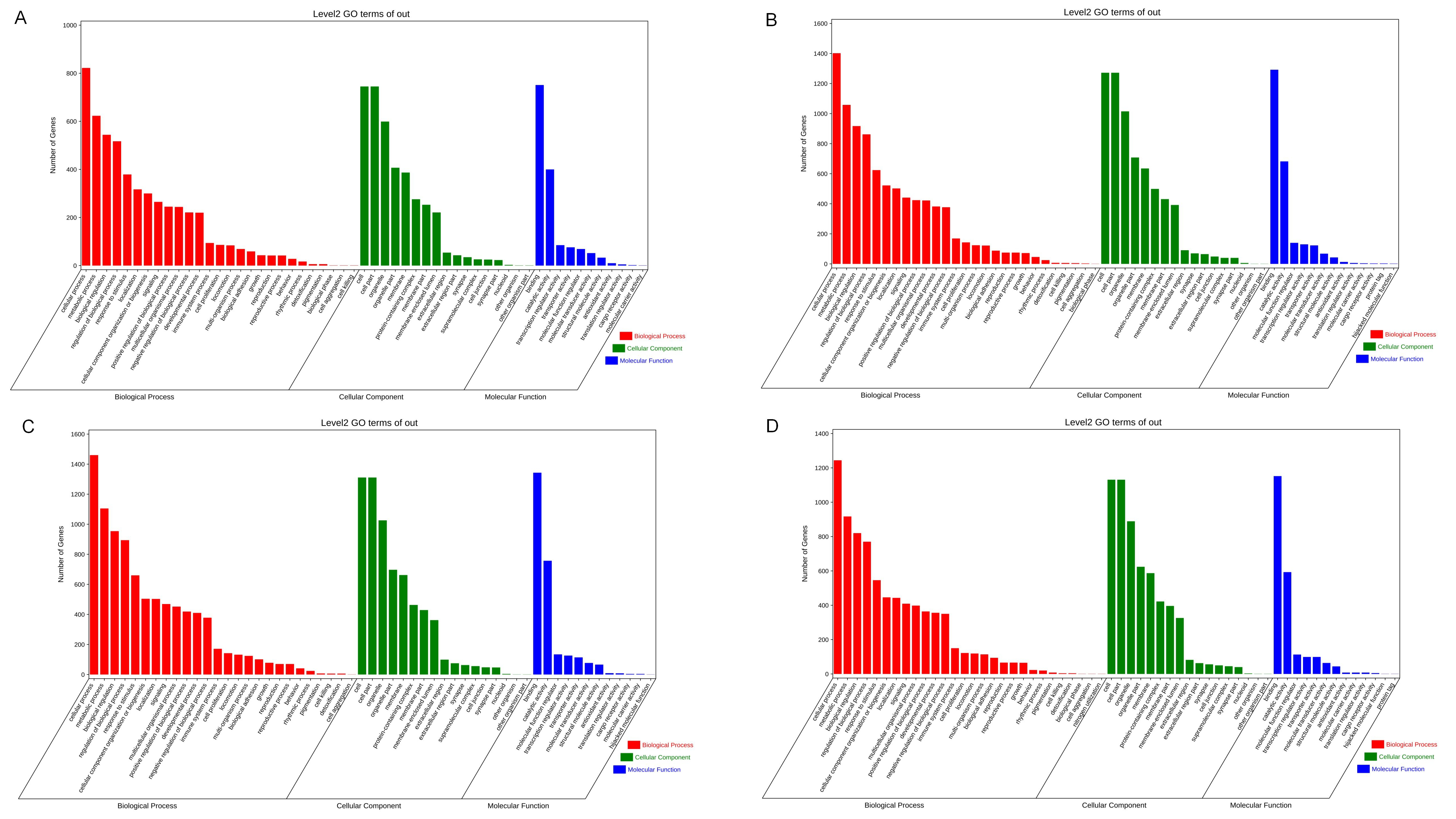

Supplement: Supplementary file 1 [file genes-15-00812-s001.zip › SM Figure/SM Figure S2. GO analysis of highly expressed differential miRNAs target genes.jpeg]
